# Leg Movement Activity During Sleep in Adults With Attention-Deficit/Hyperactivity Disorder

**DOI:** 10.3389/fpsyt.2018.00179

**Published:** 2018-05-04

**Authors:** Corrado Garbazza, Cornelia Sauter, Juliane Paul, Jenny Kollek, Catharine Dujardin, Sandra Hackethal, Hans Dorn, Anita Peter, Marie-Luise Hansen, Mauro Manconi, Raffaele Ferri, Heidi Danker-Hopfe

**Affiliations:** ^1^Competence Centre of Sleep Medicine, Charité—Universitätsmedizin Berlin, Humboldt-Universität zu Berlin, Berlin Institute of Health (BIH), Berlin, Germany; ^2^Sleep and Epilepsy Center, Neurocenter of Southern Switzerland, Civic Hospital of Lugano (EOC), Lugano, Switzerland; ^3^Department of Neurology, University Hospital of Zurich, Zurich, Switzerland; ^4^Sleep Research Centre, Department of Neurology I.C., Oasi Institute for Research on Mental Retardation and Brain Aging (IRCCS), Troina, Italy

**Keywords:** attention-deficit/hyperactivity disorder, sleep disorders, restless legs syndrome, periodic leg movements during sleep, periodicity index

## Abstract

**Objectives:** To conduct a first detailed analysis of the pattern of leg movement (LM) activity during sleep in adult subjects with Attention-Deficit/Hyperactivity Disorder (ADHD) compared to healthy controls.

**Methods:** Fifteen ADHD patients and 18 control subjects underwent an in-lab polysomnographic sleep study. The periodic character of LMs was evaluated with established markers of “periodicity,” i.e., the periodicity index, intermovement intervals, and time distribution of LM during sleep, in addition to standard parameters such as the periodic leg movement during sleep index (PLMSI) and the periodic leg movement during sleep arousal index (PLMSAI). Subjective sleep and psychiatric symptoms were assessed using several, self-administered, screening questionnaires.

**Results:** Objective sleep parameters from the baseline night did not significantly differ between ADHD and control subjects, except for a longer sleep latency (SL), a longer duration of the periodic leg movements during sleep (PLMS) in REM sleep and a higher PLMSI also in REM sleep. Data from the sleep questionnaires showed perception of poor sleep quality in ADHD patients.

**Conclusions:** Leg movements during sleep in ADHD adults are not significantly more frequent than in healthy controls and the nocturnal motor events do not show an increased periodicity in these patients. The non-periodic character of LMs in ADHD has already been shown in children and seems to differentiate ADHD from other pathophysiological related conditions like restless legs syndrome (RLS) or periodic limb movement disorder (PLMD). The reduced subjective sleep quality reported by ADHD adults contrasted with the normal objective polysomnographic parameters, which could suggest a sleep-state misperception in these individuals or more subtle sleep abnormalities not picked up by the traditional sleep staging.

## Introduction

The complex relationship between sleep and attention-deficit/hyperactivity disorder (ADHD) has been widely investigated, especially in children (1, 2). Given the high prevalence of sleep disturbances subjectively reported by these patients (2–4), researchers have been encouraged to further investigate the characteristics of sleep in ADHD by using instrumental tools, such as actigraphy and polysomnography (PSG) (2). While a number of PSG studies highlighted some differences in sleep macro- and/or microstructure between ADHD and healthy control subjects (5–8), others could not find robust evidence of a different sleep pattern in the two groups (9–13). In fact, Cohen-Zion and Ancoli-Israel, in one of the first comprehensive literature reviews on sleep in children with ADHD, already concluded that “actigraphic and PSG data have not identified clear and consistent differences in measures of sleep continuity or sleep architecture between children with and without ADHD” [(14), p. 397].

Some authors have hypothesized that the typical daytime hyperactivity observed in ADHD may be mirrored by an increased motor activity during the night, which in turn may cause sleep disruption and therefore explain the reduced sleep quality mostly experienced by these patients. Furthermore, ADHD is often associated with sleep-related movement disorders, such as restless legs syndrome (RLS) and periodic limb movement disorder (PLMD) (15–19). It has been suggested that sleep disruption associated with these disorders and the motor restlessness of RLS while awake could contribute to the typical symptoms of inattention and hyperactivity seen in ADHD children (16). Also, some authors pointed out that all these conditions may share a similar pattern of periodic leg movements during sleep (PLMS), and even a possible common pathophysiology related to a central dopaminergic dysfunction (20, 21) or brain iron deficiency (22–24).

However, again, data from the literature are not consistent, with some studies supporting the notion that sleep in ADHD is characterized by a pathologically elevated number of periodic leg movements (PLM) during sleep (PLMS) or during wakefulness (PLMW) compared to healthy subjects (15, 25–28), while other investigations do not confirm these findings (5, 9, 29).

Moreover, Bruni et al. (30) and Ferri et al. (31) showed that PLMS in ADHD children present substantially different features from those usually observed in RLS patients, since they have a low periodic character (low Periodicity Index), a scarce circadian decrement across the night, and are not responsive to *L*-DOPA treatment. Thus, the authors suggested that the generating mechanisms of PLMS in ADHD and RLS may also differ.

These findings raised new interest in studying the peculiar characteristics of leg movements (LMs) during sleep in adult ADHD using advanced measures, that have been introduced in the past years to analyse the time structure of LM activity during sleep in RLS (32) and other disorders, such as PLMD (33, 34), REM sleep behavior disorder (35), narcolepsy/cataplexy (36), and ADHD in children (31).

To date, only four objective sleep studies in ADHD adult patients have been published, of which three also considered the leg movement activity during sleep in these subjects. While Kooij et al. analyzed data from actigraphy (37), Philipsen et al. (10) and Sobanski et al. (6) used polysomnography (PSG) over 2 nights and compared the PLMS index (number of PLMS per hour of sleep—PLMSI) and the PLMS arousal index (number of PLMS per hour of sleep that are associated with an EEG arousal—PLMSAI) in ADHD and control subjects with mixed results. In fact, in the study by Philipsen (10) the PLMSI was significantly higher in 20 ADHD subjects compared to 20 controls (5.18 ± 5.92 vs. 1.66 ± 3.25, *p* = 0.005). Similar results were observed by Sobanski et al. (6) (5.3 ± 5.7) in 34 adult ADHD patients. However, the PLMSAI of ADHD patients in the first study was not only higher than in control subjects (1.56 ± 2.19 vs. 0.31 ± 0.54, *p* = 0.011) (10), but also compared to the PLMSAI of ADHD subjects examined in the second study (0.3 ± 0.4) (6). Moreover, in contrast to Philipsen et al. (10), Sobanski et al. (6) found polysomnographic differences between ADHD and controls, with patients showing a reduced sleep efficiency, as well as an elevated number of awakenings and a higher percentage of wakefulness after sleep onset. Overall, PLMSI and PLMSAI values in both studies were far from the respective pathological thresholds for healthy adult subjects (38).

The inconsistent results of these studies raise the question of whether leg movements during sleep are a common finding in ADHD, and if so, whether they show specific features of “periodicity.” In the present study, a first detailed analysis of the pattern of leg movement (LM) activity during sleep in adults with ADHD compared to gender- and age- matched healthy controls was conducted. Established markers of “periodicity,” i.e., the periodicity index, intermovement intervals, and time distribution of LM during sleep, in addition to standard parameters such as the periodic leg movement during sleep index (PLMSI) and the periodic leg movement during sleep arousal index (PLMSAI), were evaluated, to describe the periodic character of LMs. Furthermore, it was examined whether ADHD subjects differ from healthy controls with regard to objective sleep parameters.

## Materials and methods

### Subjects and screening

Twenty-two ADHD patients and 20 healthy control subjects were originally enrolled in a pilot study investigating the association between sleep and memory consolidation during sleep in adults diagnosed with ADHD according to DSM-IV (39). Patients were recruited during 2011 and 2012, in cooperation with the outpatient center for adult ADHD (“Centrum für ADHS im Erwachsenenalter”) at the Charité Medical University, Department of Psychiatry, Campus Benjamin Franklin, Berlin. ADHD diagnosis was made close to the study enrollment. Controls were recruited via an advertisement at the Freie Universität Berlin and the Charité Berlin. In particular, healthy individuals matching with ADHD subjects of the same age (± 2.5 years) and gender were specifically selected. The study was approved by the ethics committee of the Charité (EA4/011/11). All subjects gave written informed consent.

Participants were first invited to a clinical interview and a medical examination by a study physician, who excluded subjects with relevant somatic and neurologic diseases, or with a current or previous substance abuse. A series of screening questionnaires was administered to characterize sleep quality and habits of the participants, as well as possible comorbid mood and anxiety disorders.

The inclusion/exclusion criteria for ADHD and control subjects as well as the screening questionnaires used in the study are summarized in Table 1.

**Table 1:** Main inclusion and exclusion criteria for ADHD patients and control subjects considered for the analysis of leg movement activity.

	**Inclusion criteria**	**Exclusion criteria**
ADHD patients	Diagnosis of ADHD in adulthood (according to DSM-IV criteria) Male or female subjects between 18 – 60 years Sufficient speech comprehension	No capacity to consent Cognitive impairment Clinically relevant somatic and neurologic diseases (including RLS) Irregular sleep-wake rhythm (e.g. shift work) Substance abuse AHI > 15/h
Control group	Male or female subjects between 18 and 60 years Sufficient speech comprehension No conditions or medications influencing sleep, wakefulness or memory consolidation No reduced sleep quality (PSQI ≤ 5), depressive or anxiety disorders (SDS < 40, SAS < 36) or increased daytime sleepiness (ESS ≤ 10)	No capacity to consent Cognitive impairment Clinically relevant somatic and neurologic diseases (including RLS) Substance abuse Irregular sleep-wake rhythm (e.g., shift work) AHI > 15/h

*PSQI, Pittsburgh Sleep Quality Index (40); SAS, Self-Rating Anxiety Scale (41, 43); SDS, Self-Rating Depression Scale (43, 42); ESS, Epworth Sleepiness Scale (44); AHI, Apnea-hypopnea index; RLS, restless legs syndrome*.

Although suffering from comorbid RLS was not an exclusion criterion for ADHD subjects to participate in the primary (pilot) study, we decided not to consider patients and controls with a previous or current diagnosis of RLS for the analysis of leg movements during sleep (*n* = 1 ADHD subject, no controls excluded). The study physician screened all individuals for RLS based on medical history and by applying the Restless-Legs-Diagnose-Index (RLS-DI), which includes the essential diagnostic criteria established by the International RLS Study Group (45). In fact, we intended to examine the characteristics of the nocturnal motor activity in a sample of adult ADHD, whose sleep was not influenced by other conditions, that are known to be associated with an elevated amount of PLMS, such as RLS. Furthermore, we excluded subjects with an apnea-hypopnea index (AHI)>15 (*n* = 2 ADHD subjects; none of the control subjects) because of the possible confounding role of respiratory-related leg movements with respect to the total amount of PLMS (46). Two ADHD patients declined to participate after the adaptation night. After exclusion of 2 individuals from each group for technical reasons related to electromyogram (EMG) recording from the lower limbs, leg movement activity was secondarily analyzed in age- and gender- balanced groups of 15 ADHD patients (33.9 ± 7.9 years.; *m* = 6, *f* = 9) and 18 healthy controls (35.8 ± 7.5 years.; *m* = 8, *f* = 10). All participants were Caucasian.

### Polysomnographic recordings

According to the study protocol, all participants (ADHD and controls) attended two full-night polysomnographic recordings. The first night served as an adaptation to the sleep laboratory environment and a screening for sleep disordered breathing (AHI > 15/h). This was consecutively followed by a baseline night, which provided the data presented in this paper. The polysomnographic recording montage included electroencephalogram (EEG) (F3, F4, C3, C4, O1, O2, CZ, and A1, A2 as reference electrodes on the mastoid), horizontal and vertical electrooculogram (EOG), electromyogram (EMG) from the surface of the submentalis muscle (3 electrodes), the right and left tibialis anterior muscles (2 electrodes), tracheal microphone, oro-nasal airflow, thoracoabdominal respiratory movements, electrocardiogram (ECG), and oxyhaemoglobin saturation (SaO2) by means of finger oximeter.

### Sleep scoring and analysis of LMs

The recordings were submitted to an external and independent competence center for sleep analysis (The Siesta Group Schlafanalyse GmbH, Vienna), which provided a validated and FDA approved sleep scoring under expert human supervision (47) according to the American Academy of Sleep Medicine (AASM) standard criteria (48).

Leg movement activity during sleep was recorded using electrodes placed bilaterally on the tibialis anterior muscles, according to standard recommendations (48). Leg movements were first automatically detected by the software Hypnolab 1.2 (SWS Soft, Italy), which is a validated, computer-assisted system (49), using an automatic approach under human supervision. One scorer (CG) visually edited the detections proposed by the software before computing a final result. The scorer was not blinded to the group the participants belonged to (ADHD vs. controls), since the same person was also responsible for the study recruitment and conduct. PLM were identified according to the criteria of the International Restless Legs Syndrome Study Group (IRLSSG), endorsed by the World Association of Sleep Medicine (WASM) (50). Besides the standard indexes (50), the following additional parameters were calculated:

1. The Periodicity Index (PI), which, as defined by Ferri et al. (32), is computed as the ratio between the number of intermovement intervals contained in regular uninterrupted sequences of at least 4 LMs, separated by 10–90 s intervals (onset-to-onset) and the total number of intermovement intervals recorded. This index can vary between 0 (absence of periodicity) and 1 (all intervals with length 10 < i ≤ 90 s) and is independent of the absolute number of LMs recorded. A high PI indicates a high level or order of LMs, while a low PI stands for a high level of entropy of the leg motor activity during sleep or wakefulness (32)
2. The intermovement intervals distribution (IMI), which in RLS subjects shows a bimodal pattern, with a first, smaller peak at around 4 s and a second main peak at around 20–25 s, mostly corresponding to PLMS (32)
3. The distribution of PLMS across the night, which in adult RLS subjects follows a circadian trend with a progressive reduction across the night and peaks with the nadir in body temperature (33)
4. The alternative PLMS index, which only considers LMs separated by 10–90 s and series of PLMS not interrupted by LMs with inter-LM interval shorter than 10 s, was calculated based on previous research performed by Ferri et al. (51, 52). This index has been shown to better describe the genuine periodic leg movement activity during sleep than the standard PLMS index.

### Statistical data analysis

Statistical analysis was performed using R Statistical Software version 3.0.3 (2014) (53). For categorical data, between-group differences were assessed using the Chi-Squared test or the Student's *t*-test. All remaining comparisons were carried out using the Mann–Whitney test for independent data samples. Differences were reported as significant when they reached a level of *p* < 0.05 (two-sided). *P*-values were corrected using Holm's correction separately for each cluster (i.e. screening questionnaires, sleep parameters, leg movement activity parameters, distribution of intermovement intervals during sleep and number of isolated and periodic LMs per hour of sleep). As a measure of effect size, we additionally reported the common language effect size for non-parametric testing. First introduced by McGraw and Wong (54), the common language effect size is a statistic which has a more intuitive interpretation than most other effect size measures. In our case, it represents the probability (%) that a randomly selected data case of the control group will have a higher value when compared to a randomly selected data case of the ADHD subjects within the same variable. For normal distribution the statistic is calculated as m(x)−m(y)/sqrt(s^2^(x)+s^2^(y)), where m is the mean and s^2^ is the variance of the two variables x and y, and then determining the probability associated to the resulting z score. Since our data violated the assumption of normality, we calculated the Common Language as a non-parametric effect size estimation as follows: 1-U/n(x)n(y), where U is the statistic calculated by the Mann–Whitney-*U*-test and *n* is the sample size.

## Results

### Questionnaire data

The results of the screening questionnaires and the questionnaires for the characterization of the sample submitted to the participants on the adaptation night are reported in Table 2.

**Table 2:** Descriptive statistics for age, BMI, the scores of the screening questionnaires, and results of the test for differences between ADHD subjects and controls.

	**ADHD patients (*****n*** = **15)**		**Control group (*****n*** = **18)**		
	**Mean ±*SD***	**Median (IQR)**	**Mean ±*SD or* n**	**Median (IQR)**	**Corrected *p-*values**
Age	33.9 ± 7.9	32 (27; 41)	35.8 ± 7.5	35.8 (30.8; 44.3)	0.758^a^
BMI	24.7 ± 2.6	24.6 (22.7; 26.4)	24.0 ± 2.3	24.2 (22.1; 26.0)	0.758^b^
PSQI	7.5 ± 2.7	7.0 (5.5; 10)	3.4 ± 1.4	4.0 (3.0; 4.0)	<0.001^a^
ISI	11.3 ± 7.2	12.0 (6.0; 15.5)	3.2 ± 3.7	2.0 (1.0; 4.0)	0.002^a^
ESS	7.3 ± 4.7	7.0 (4.5; 8.0)	5.4 ± 3.2	5.0 (2.0; 8.0)	0.372^a^
SAS	34.7 ± 6.9	34.0 (30.0; 39.0)	25.0 ± 3.5	24.5 (22.0; 27.0)	<0.001^a^
SDS	41.7 ± 7.7	44.0 (37.5; 45.5)	27.2 ± 5.2	26.0 (23.0; 30.0)	<0.001^a^
	*n* (%)		*n* (%)		
MEQ (> 58)	1 (6.7%)		9 (50.0%)		0.052^c^
MEQ (42-58)	8 (53.3%)		5 (27.8%)		
MEQ (< 42)	6 (40.0%)		4 (22.2%)		

*BMI, Body Mass Index; ESS, Epworth Sleepiness Scale (44); PSQI, Pittsburgh Sleep Quality Index (40); SAS, Self-Rating Anxiety Scale (41); SDS, Self-Rating Depression Scale (42); ISI, Insomnia Severity Index (55); MEQ, Morningness-Eveningness Questionnaire (56) (MEQ > 58: morning chronotype; MEQ 42–58: indifferent chronotype; MEQ < 42: evening chronotype)*.

a Mann–Whitney U-test;

b Student's t-test;

c *Chi-squared test*.

Mean daytime sleepiness, assessed by the Epworth Sleepiness Scale (ESS), did not differ between the groups, with only 3 patients having an ESS score over the pathological threshold of 10. The Pittsburgh Sleep Quality Index (PSQI) total score, as well as the mean scores of Self-Rating Anxiety Scale (SAS) and Self-Rating Depression Scale (SDS) were significantly higher in ADHD, as expected, according to the inclusion criteria for control subjects. In particular, 11 of 15 patients had a PSQI total score > 5, while the scores of SAS and SDS in ADHD were still within the normal range (41–43). The Insomnia Severity Index (ISI) was also higher in ADHD with 9 patients vs. 3 controls having an ISI score > 7, indicating a higher prevalence of insomnia symptoms in the first group.

Finally, the results from the Morningness–Eveningness Questionnaire (MEQ) were different between the two groups, with a higher number of evening and indifferent chronotypes (40.0 vs. 22.2% and 53.3 vs. 27.3%, respectively) and a lower number of morning chronotypes (6.7 vs. 50.0%) observed in ADHD compared to control subjects, but with overall no significant differences between groups.

### Polysomnography data

An overview of the sleep and leg movement activity parameters analyzed in the study is reported in Tables 3, 4. PSG data pertaining to the sleep structure and based on the standard AASM parameters (48) did not show significant differences between ADHD patients and control subjects, with the exception of the sleep latency (SL), which was increased in ADHD (*p* = 0.007).

**Table 3:** Sleep parameters in ADHD subjects and controls.

	**ADHD (*****n*** = **15)**		**Controls (*****n*** = **18)**		***Mann-Whitney-U p***	**Common language effect size (%)**
	**Mean ±*SD***	**Median (IQR)**	**Mean ±*SD***	**Median (IQR)**		
Recording time (TIB), min	480.5 ± 1.5	480.0 (480.0; 480.0)	480.3 ± 0.7	480.0 (480.0; 480.0)	0.919	50.9
Total sleep period (TSP), min	460.1 ± 12.8	465.5 (449.3; 469.5)	464.8 ± 12.7	467.3 (462.5; 473.0)	0.329	60.2
Total sleep time (TST), min	429.6 ± 32.4	435.0 (417.8; 454.0)	424.7 ± 38.7	432.5 (413.5; 445.5)	0.745	46.5
Sleep latency (N1), min	21.3 ± 12.2	19.5 (13.0; 30.3)	10.8 ± 7.9	9.0 (3.5; 17.5)	0.007^*^	22.4
REM sleep latency, min	103.2 ± 43.9	94.5 (68.5; 129.3)	80.8 ± 21.7	79.0 (62.5; 89.0)	0.181	36.1
Stage shifts/h	19.5 ± 5.1	19.0 (16.2; 23.2)	21.2 ± 5.6	19.6 (17.1; 25.7)	0.504	57.0
Awakenings/h	2.1 ± 1.0	1.9 (1.3; 2.7)	2.5 ± 1.1	2.7 (1.6; 3.1)	0.218	62.8
Sleep efficiency, %	89.4 ± 6.7	90.4 (86.9; 94.0)	88.4 ± 8.1	90.0 (86.2; 92.8)	0.786	47.0
Wake after sleep onset (WASO), %	30.0 ± 26.7	21.0 (14.3; 32.3)	44.9 ± 38.6	29.8 (21.5; 59.0)	0.138	65.4
**SLEEP STAGES**						
N1, %	11.5 ± 5.0	10.8 (8.1; 16.2)	10.8 ± 3.7	9.8 (9.4; 11.6)	0.772	46.9
N2, %	42.5 ± 6.6	40.7 (38.6; 45.8)	46.7 ± 7.4	46.6 (41.6; 49.8)	0.076	68.3
N3, %	26.7 ± 11.8	27.0 (19.8; 31.6)	22.5 ± 7.8	21.1 (17.6; 29.6)	0.347	40.2
REM, %	19.3 ± 4.9	20.0 (17.8; 21.3)	19.9 ± 3.1	20.5 (17.3; 22.9)	0.718	53.9
Arousal index (AI), total sleep	19.3 ± 9.1	17.0 (13.1; 24.2)	19.3 ± 8.2	17.6 (13.5; 24.3)	0.942	50.9
Arousal index (AI), NREM	22.3 ± 10.3	19.5 (16.3; 27.7)	22.5 ± 10.3	18.6 (15.7; 29.5)	0.971	49.4
Arousal index (AI), REM	6.1 ± 5.7	4.2 (2.1; 8.0)	6.8 ± 4.9	4.4 (3.5; 7.9)	0.437	58.1
AHI	3.0 ± 3.0	1.9 (0.9; 3.7)	2.2 ± 3.4	1.1 (0.3; 2.1)	0.148	35.0

*N1, N2, N3, NREM sleep stages 1, 2, and 3; R, REM sleep stage; NREM, non-rapid eye movement sleep; REM, rapid eye movement sleep*.

* *p < 0.05*.

**Table 4:** Leg movement activity parameters in ADHD subjects and controls.

	**ADHD (*****n*** = **15)**		**Controls (*****n*** = **18)**		***Mann-Whitney-U p***	**Common language effect size (%)**
	**Mean ±*SD***	**Median (IQR)**	**Mean ±*SD***	**Median (IQR)**		
**TOTAL SLEEP**						
Total index	12.0 ± 8.1	6.7 (2.8; 10.9)	8.3 ± 6.8	6.7 (2.8; 10.9)	0.104	33.1
PLMS index	5.1 ± 7.2	1.3 (0.0; 2.5)	3.0 ± 4.7	1.3 (0.0; 2.5)	0.365	40.6
Isolated LM index	6.9 ± 2.3	5.3 (2.8; 7.5)	5.3 ± 2.9	5.3 (2.8; 7.5)	0.093	32.6
**NREM**						
Total index	11.9 ± 10.5	10.9 (5.8; 13.9)	8.1 ± 7.7	6.1 (1.7; 10.6)	0.215	37.0
PLMS index	5.9 ± 9.6	3.4 (0.7; 5.2)	3.4 ± 6.0	1.2 (0.0; 3.1)	0.512	43.1
Isolated LM index	6.0 ± 2.1	6.0 (5.3; 9.2)	4.7 ± 2.7	4.6 (1.7; 6.9)	0.153	35.2
**REM**						
Total index	13.3 ± 6.5	11.6 (8.2; 17.1)	9.5 ± 7.9	8.2 (4.0; 13.8)	0.073	31.5
PLMS index	2.8 ± 2.9	2.5 (0.0; 4.2)	1.3 ± 3.3	0.0 (0.0; 0.5)	0.044^*^	30.9
Isolated LM index	10.5 ± 4.9	8.5 (7.4; 12.0)	8.1 ± 5.3	8.2 (3.8; 11.1)	0.215	37.0
**PLMS ANALYSIS**						
PLMS sequence number	3.6 ± 3.0	2.0 (1.0; 6.5)	2.3 ± 2.5	2.0 (0.0; 3.0)	0.262	38.5
PLMS sequence duration, s	281.0 ± 302.2	160.8 (84.3; 242.0)	245.8 ± 213.0	150.7 (121.5; 267.1)	0.867	73.3
PLMS duration in NREM, s	2.0 ± 1.1	2.0 (1.5; 2.5)	2.3 ± 1.1	2.0 (1.6; 3.0)	0.808	45.3
PLMS duration in REM, s	1.4 ± 1.2	1.8 (0.0; 2.1)	0.5 ± 0.8	0.0 (0.0; 1.3)	0.023^*^	17.9
Isolated LM duration in NREM, s	3.00 ± 0.9	2.7 (2.6; 3.2)	2.9 ± 1.0	2.9 (2.3; 3.4)	0.986	49.6
Isolated LM duration in REM, s	2.3 ± 1.0	2.0 (1.9; 2.6)	2.6 ± 1.5	2.2 (1.6; 3.6)	0.638	55.0
Periodicity index, total	0.2 ± 0.2	0.1 (0.0; 0.2)	0.1 ± 0.2	0.1 (0.0; 0.2)	0.926	51.1
Periodicity index, NREM	0.2 ± 0.3	0.1 (0.0; 0.3)	0.2 ± 0.2	0.1 (0.0; 0.2)	0.924	48.9
Periodicity index, REM	0.0 ± 0.1	0.0 (0.0; 0.0)	0.0 ± 0.1	0.0 (0.0; 0.1)	1.000	50.2
Alternative PLMS index, total	3.7 ± 6.8	0.9 (0.0; 3.4)	2.2 ± 4.3	0.6 (0.0; 1.3)	0.725	46.3
Alternative PLMS index, NREM	4.6 ± 9.0	1.0 (0.0; 3.8)	2.6 ± 5.6	0.3 (0.0; 1.5)	0.645	45.4
Alternative PLMS index, REM	0.7 ± 1.4	0.0 (0.0; 0.8)	0.7 ± 1.2	0.0 (0.0; 2.0)	0.818	52.0
PLMS/arousal index, total sleep	1.6 ± 1.8	1.5 (0.4; 2.2)	0.9 ± 1.1	0.6 (0.1; 1.1)	0.364	40.6
PLMS/arousal index, NREM	1.8 ± 2.0	1.7 (0.4; 2.2)	1.1 ± 1.3	0.7 (0.2; 1.4)	0.373	40.7
PLMS/arousal index, REM	0.7 ± 1.1	0.0 (0.0; 1.0)	0.2 ± 0.5	0.0 (0.0; 0.0)	0.112	37.0

*N1, N2, N3, NREM sleep stages 1, 2, and 3; R, REM sleep stage; NREM, non-rapid eye movement sleep; REM, rapid eye movement sleep; LM, leg movements; PLMS, periodic leg movements during sleep*.

* *p < 0.05*.

Data from the analysis of leg movements during sleep also did not show any significant differences, apart from a slightly higher total index of PLMS (*p* = 0.044; CL = 30.9) and a longer PLMS duration (*p* = 0.023; CL = 17.9), both in REM sleep (Table 4). Again, none of these differences remained when correcting for multiple testing. The results of the Common Language effect size also did not point toward any obvious trends within the data for the analyzed comparisons.

Regarding the periodic pattern of LMs, not only standard parameters, like the PLMS index and PLMS arousal index, but also more specific “detectors of periodicity,” such as the Periodicity Index (PI) and the alternative PLMS index were found to not differ statistically between the groups. Although the PLMSI was higher in ADHD subjects, this was not significantly different from controls and clearly below the threshold of clinical relevance (PLMSI > 15) (38). The same applied to the PLMSAI, the PI, and the alternative PLMSI.

**Figure 1:**
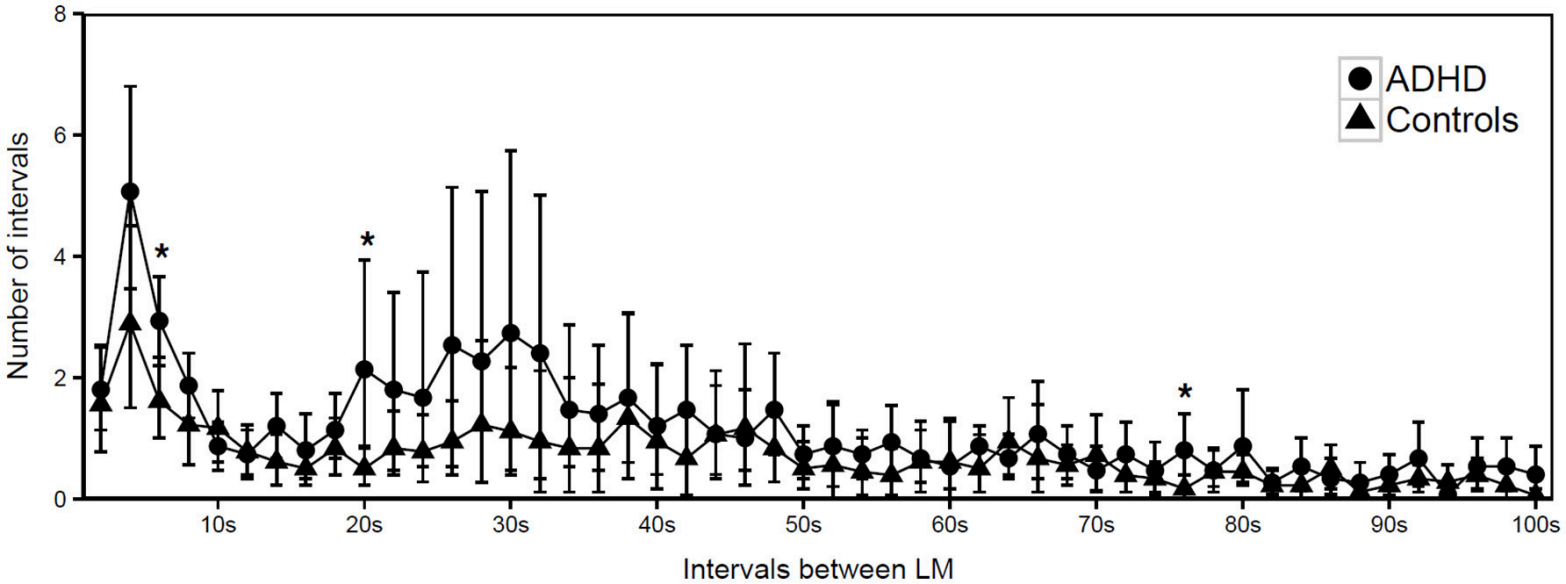
Distribution of intermovement intervals during sleep in participants with ADHD and controls (mean and standard deviation). ^*^p < 0.05.

**Figure 2:**
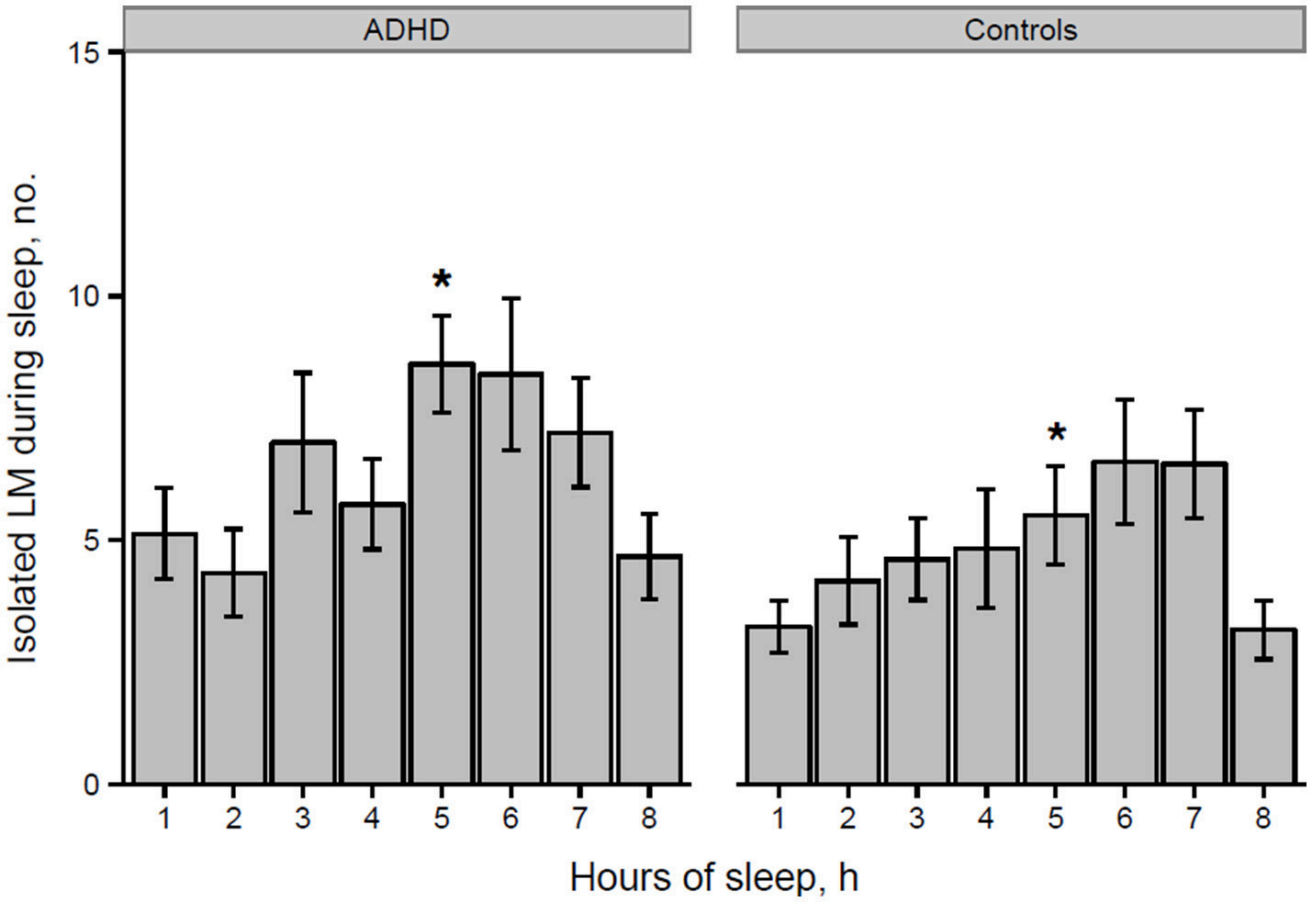
Number of isolated LMs per hour of sleep in participants with ADHD and controls (*p = 0.028 at hour 5 of sleep). ^*^p < 0.05.

**Figure 3:**
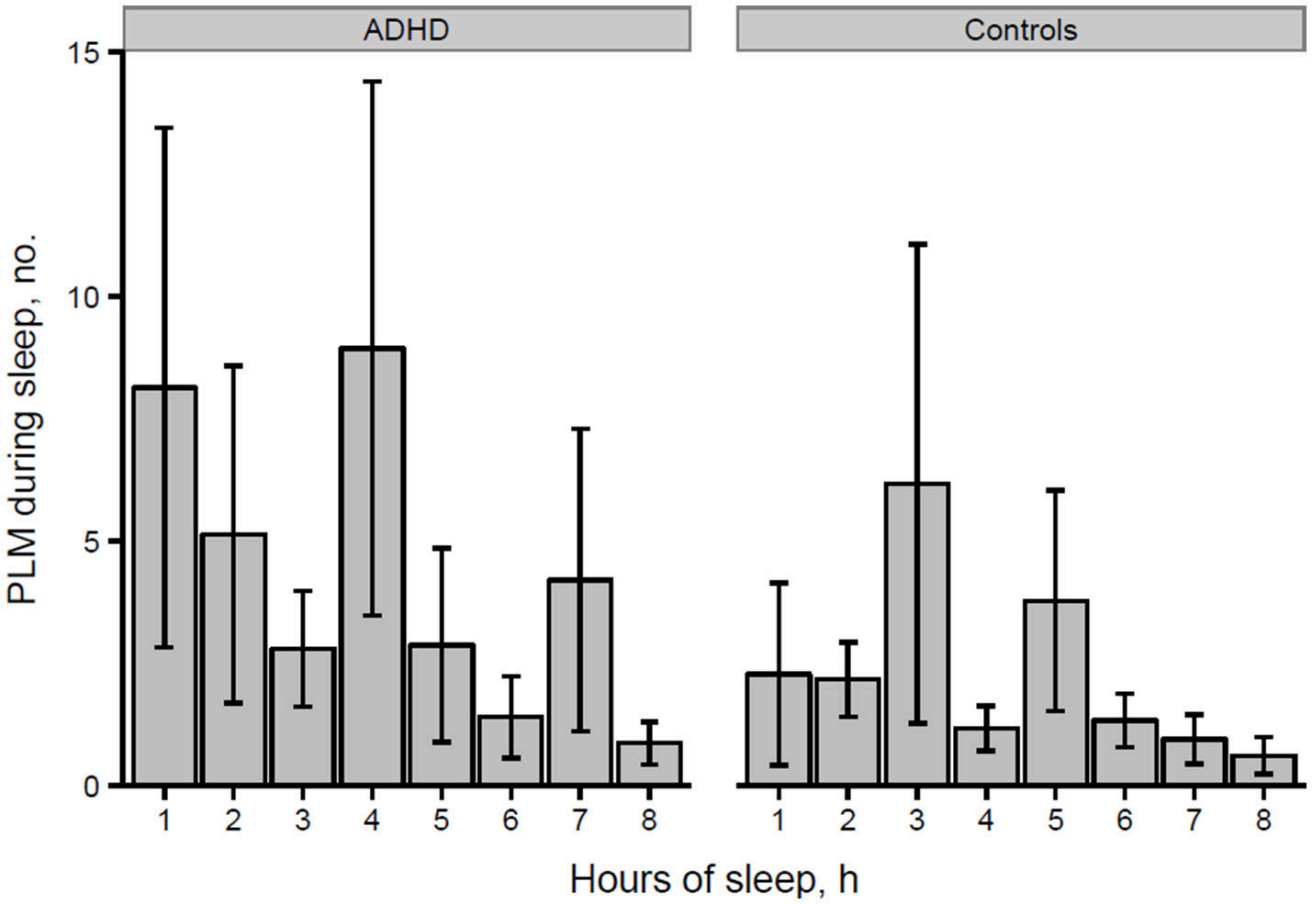
Number of PLMS per hour of sleep in participants with ADHD and controls.

However, the distribution histogram of inter-LM intervals (Figure 1) tended to show higher values in ADHD than in controls during both the early peak with intervals <10 s and in the typical range of PLMS (14–42 s in this case), but only reached statistical significance at 4–6 s (*p* = 0.022), 18–20 s (*p* = 0.048), and 74–76 s (*p* = 0.034). By contrast, the only ADHD subject, who was also affected by RLS and therefore not considered for the leg movement analysis, showed a clearly prominent peak at 8–30 s, corresponding to the typical pattern of inter-LM intervals distribution seen in RLS patients (32) (see Supplementary Image 1). LMs were quite irregularly distributed across the night in both groups of subjects (Figures 2, 3) and there were no consistent differences.

Finally, when considering a correction for multiple comparisons carried out within this same data set, none of the above mentioned between-group differences would reach statistical significance (see Statistical Data Analysis).

### Medications and psychiatric comorbidities of the study participants

With regard to the pharmacological treatments received by the ADHD patients, four subjects were taking methylphenidate and one atomoxetine. Two subjects were also diagnosed with major depressive disorder and one of them was treated with sertraline and quetiapine. Three patients were affected by comorbid recurrent major depressive disorder, currently in remission, and one of them, who additionally presented agoraphobia and panic disorder, was treated with citalopram. One subject was taking fluoxetine because of a concomitant diagnosis of dysthymia. None of the patients was taking benzodiazepines and seven did not have any medication. Also, none of the control subjects was treated with any psychotropic drugs.

## Discussion

The aim of the present study was to conduct a first detailed analysis of the pattern of leg movement activity during sleep in ADHD adults compared to healthy controls. The main result is a lack of significant differences in standard sleep and movement parameters, as well as advanced periodicity markers between adult ADHD and control subjects.

There have been only two previous polysomnographic studies on leg movement activity in adult ADHD (6, 10), and these have focused on few standard parameters such as the PLMSI and the PLMSAI, reporting contrasting results, as discussed in the section Introduction. The accuracy of PLMSI and PLMSAI as markers of “periodicity” has been debated after Ferri et al. (32) introduced more specific parameters (Periodicity Index, inter-LM interval, and time distribution of LMs through the night), which have been shown to better “capture” the periodic character of LMs, and also suggested an alternative PLMS index, which was more reliable in detecting genuinely periodic LMs (51, 52).

In our analysis both the standard and alternative PLMS index, the Periodicity Index, as well as the PLMSAI were not significantly different between adult ADHD subjects and healthy controls. In particular, the PLMSI per hour of total sleep time (TST) in ADHD was very similar to that found by Philipsen (10) and Sobanski (6), but not significantly higher than in the control group and, as in the previous studies, not clinically relevant.

The alternative PLMS index was even lower than the standard PLMS index, as expected, confirming the low periodic character of LMs in these subjects. The periodicity index (PI) was also low, both in ADHD and controls, as already observed in children (31), and clearly lower than the values found in other sleep disorders such as adult RLS, narcolepsy, rapid eye movement sleep behavior disorder, and in control subjects >40 years of age (32, 35, 36, 57, 58).

The intermovement intervals distribution (IMI) histogram (Figure 1) was characterized by a prominent peak on the left side of the graph, at ~4 s, which has already been observed studying LM activity during sleep in ADHD children (31) and depends on the prevalent low periodicity of LMs in these patients. A less pronounced peak, essentially due to 2 ADHD subjects in our group (see Supplementary Image 2) with a higher PLMS index (16.1 and 26.3, respectively) and Periodicity Index (0.5 and 0.7, respectively), was present in the middle part of the histogram (interval range 14–42 s), representing the less-common, genuinely periodic LMs in ADHD.

Finally, we analyzed the distribution of the number of LMs per hour of sleep over the night, similarly to what Ferri et al. (31) performed in ADHD children and control subjects, without finding any significant differences.

Therefore, based on our results, PLMS do not seem to be a distinctive characteristic of ADHD adults. Although previous studies showed that the nocturnal motor activity may be increased in these patients (10), the absence of a periodic pattern of LM during sleep seems to clearly differentiate ADHD from other pathophysiologically related conditions, such as RLS and PLMD, possibly because the dopaminergic mechanism may not be primarily involved in ADHD (31). This hypothesis, which has already been postulated by Bruni et al. and Ferri et al. regarding ADHD children (30, 31), is confirmed by our data in adult patients.

Moreover, we did not find an evident correspondence between the subjective sleep disturbances reported by our ADHD patients when completing the sleep questionnaires (especially the ISI and PSQI, see Table 2) and objective PSG indicators of poor sleep quality. This apparent discrepancy has already been underlined by Philipsen et al. (10, 59), who mentioned the question of a “sleep-state misperception” and speculated that the cause of sleep complaints in these subjects may be related to sleep onset difficulties. In fact, in our study only the sleep latency was significantly longer in ADHD adults, as previously reported in children (31). This in turn, has been linked, by some authors, to an increased prevalence of sleep onset insomnia due to a delayed dim light melatonin onset (DLMO) in these patients (16, 60, 61) and encouraged some successful chronotherapeutic approaches with melatonin (62–64) and bright light therapy (65). In fact, almost all the ADHD patients included in our study were indifferent or evening chronotypes, as assessed by the MEQ, with only two subjects classifiable as a “morning type.”

Our findings are also overall in line with a recent meta-analysis of polysomnographic studies performed by Baglioni et al. who concluded that ADHD, together with seasonal affective disorder, is the only mental disorder with no evidence for alterations in each sleep variable and sleep domain (66). Also, Díaz-Román et al. in another systematic review and meta-analysis on sleep features in children with ADHD (specifically excluding those affected by primary sleep disorders) only found these to spend more time in sleep stage 1 than controls and suggested that the discrepancies between the findings of previous studies may be due to the large heterogeneity of the investigated populations, e.g., regarding the participants' medications and comorbid conditions, the ADHD subtypes and the assessment of disease itself (67).

Some limitations of the present study need to be discussed. Due to the small sample of subjects investigated, one could argue that this may explain the lack of differences between groups. Furthermore, the patient group comprised highly selected patients coming from a single outpatient clinic of the Charité and fulfilling the inclusion criteria of the study, thus likely to be not representative of the general adult ADHD population. Therefore, we have quantified these differences in units of a non-parametric effect size measure, which showed that our results were not due to a low statistical power related to our sample size, also considering our effect size measure.

Some of the ADHD patients had psychiatric comorbidities and were medicated. In particular, three patients were taking SSRI antidepressants, which are known to cause PLMS (68), but only one of them had a PLMSI slightly over the pathological threshold of 15/h (indeed 16.3/h). Four patients were treated with methylphenidate (MPH), which has been shown to not significantly impact on sleep architecture in a sample of ADHD children (69) and led to a reduction of sleep onset latency and an improved sleep efficiency, but not of leg movement activity, in a polysomnographic study on adult ADHD (6). As a general remark, psychiatric comorbidities are highly prevalent among adult ADHD, with estimated rates of 65–89% of patients suffering also from other psychiatric conditions, such as mood and anxiety disorders, substance use and eating or personality disorders (70, 71). Moreover, Attention-Deficit/Hyperactivity Disorder is mostly diagnosed and already treated during childhood/adolescence and persists into adulthood. Thus, although studying adult ADHD subjects with first diagnosis and who are also drug-naïve may be desirable for scientific purposes, the recruitment of such subjects may be quite challenging in a clinical research setting and not represent the real clinical situation of most adult ADHD patients. Nevertheless, the psychiatric comorbidities present in our sample of ADHD patients, which are mirrored by the scores of the SAS and SDS scales, must also be considered when looking at the results of the sleep questionnaire (i.e., PSQI, ISI), since they may account for the poor sleep quality subjectively reported by our ADHD subjects.

In conclusion, this study provides new data obtained from a detailed analysis of leg movements during sleep in ADHD adults not affected by other comorbid sleep disorders. The results contribute to the discussion whether PLMS are describing findings of ADHD in adults and if their presence may be of clinical importance. In particular, they support the hypothesis that a low periodic character of LMs seems to be typical of this condition, as already postulated for children (31) and different from other pathophysiologically related disorders such as RLS and PLMD. Also, in consideration of the overall normal sleep structure found in our group of patients, LM activity during sleep in ADHD subjects cannot explain the subjective sleep disturbances commonly reported by these patients.

Due to the relatively small number of subjects considered in this study, larger samples are needed to further confirm our observation and add details on the sleep architecture of these patients which might me disturbed by more subtle alterations not picked up by the current sleep staging methods, as already shown in ADHD children (7, 72).

## Author contributions

All authors certify that they have participated sufficiently in the work to take public responsibility for the content, including participation in the concept, design, analysis, writing, or revision of the manuscript. Furthermore, each author certifies that this material or similar material has not been and will not be submitted to or published in any other publication. The specific contributions made by each author to the submitted manuscript are indicated as follows: HD-H and CS: conception and design of study; CG, JP, CD, JK, AP, and M-LH acquisition of data (laboratory or clinical); CG, JP, HD, HD-H, CS, SH, RF, and MM: data analysis and/or interpretation; CG, CS, JP, JK, CD, SH, HD, AP, M-LH, MM, RF, and HD-H: drafting of manuscript and/or critical revision; CG, CS, JP, JK, CD, SH, HD, AP, M-LH, MM, RF, and HD-H: approval of final version of manuscript.

### Conflict of interest statement

The authors declare that the research was conducted in the absence of any commercial or financial relationships that could be construed as a potential conflict of interest.

## Supplementary material

Supplementary Image 1 Intermovement intervals of a single ADHD patient (female, age 35, under treatment with methylphenidate), who was originally included in the main study, and subsequently excluded from our analysis of leg movement activity during sleep, because of a prior diagnosis of RLS. The graph shows a prominent peak at 10–20 s, which is in the typical range for RLS patients (37, 51) and corresponds to the high periodicity commonly seen in these subjects. Periodicity Index (PI): 0.773. PLMS index: 67.58. PLMS alternative index: 57.2.

Supplementary Image 2 Distribution of intermovement intervals in individuals with ADHD. In the patient group, several subjects showed a prominent peak in the left part of the graph, which was significantly different from controls at ~4 s and indicates a higher prevalence of non-periodic LMs in ADHD. Conversely, only two patients (AD027, taking Sertralin and Seroquel, and AD039, non-medicated) showed any peak in the middle part of the graph, in particular in the 20–40 s range, which appeared more concentrated in these two ADHD subjects compared to control subjects (see Supplementary Image 3).

Supplementary Image 3 Distribution of intermovement intervals in control subjects. Healthy controls showed overall less prominent peaks in the left part of the graph, and only two subjects (KG081, KG096) showed any peak in the middle part of the graph, in particular in the 20–40 s range, which appeared less concentrated than in two ADHD subjects (see Supplementary Image 2).
