# Supplementary figures and images for: Leg Movement Activity During Sleep in Adults With Attention-Deficit/Hyperactivity Disorder
Source: Front Psychiatry. 2018 May 4;9:179. doi: 10.3389/fpsyt.2018.00179 (PMC5945883; doi:10.3389/fpsyt.2018.00179)

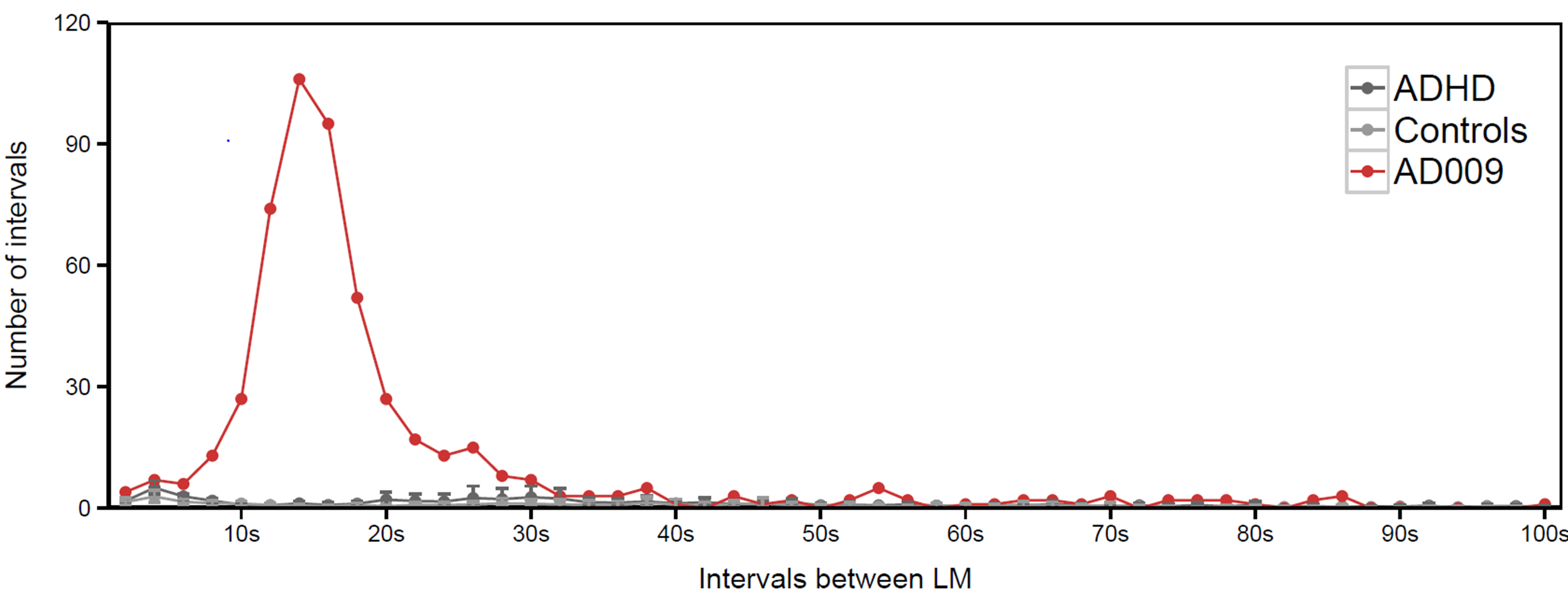

Supplement: Supplementary Image 1 — Intermovement intervals of a single ADHD patient (female, age 35, under treatment with methylphenidate), who was originally included in the main study, and subsequently excluded from our analysis of leg movement activity during sleep, because of a prior diagnosis of RLS. The graph shows a prominent peak at 10–20 s, which is in the typical range for RLS patients (37, 51) and corresponds to the high periodicity commonly seen in these subjects. Periodicity Index (PI): 0.773. PLMS index: 67.58. PLMS alternative index: 57.2. [file Image_1.TIF]

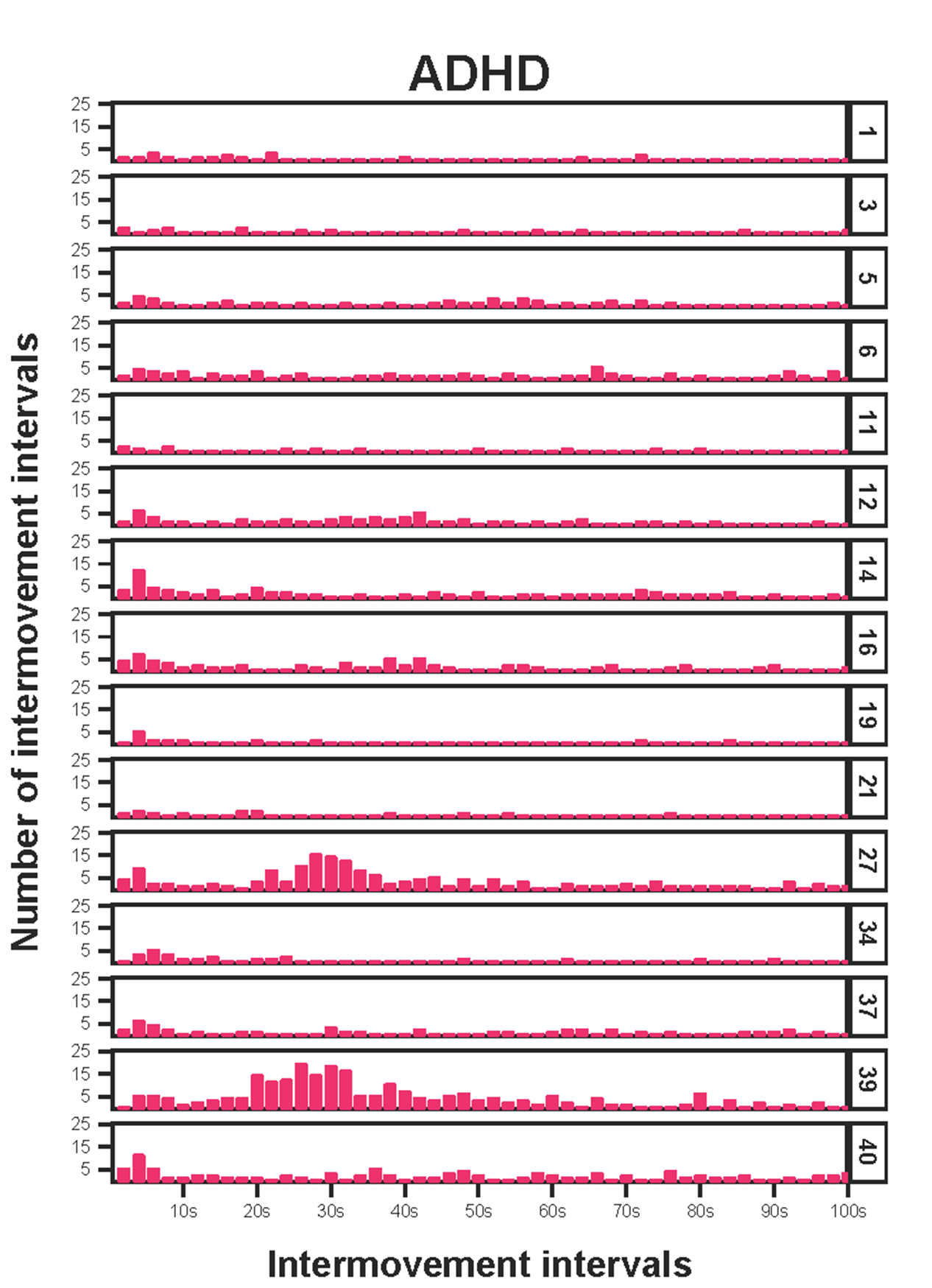

Supplement: Supplementary Image 2 — Distribution of intermovement intervals in individuals with ADHD. In the patient group, several subjects showed a prominent peak in the left part of the graph, which was significantly different from controls at ~4 s and indicates a higher prevalence of non-periodic LMs in ADHD. Conversely, only two patients (AD027, taking Sertralin and Seroquel, and AD039, non-medicated) showed any peak in the middle part of the graph, in particular in the 20–40 s range, which appeared more concentrated in these two ADHD subjects compared to control subjects (see Supplementary Image 3). [file Image_2.tif]

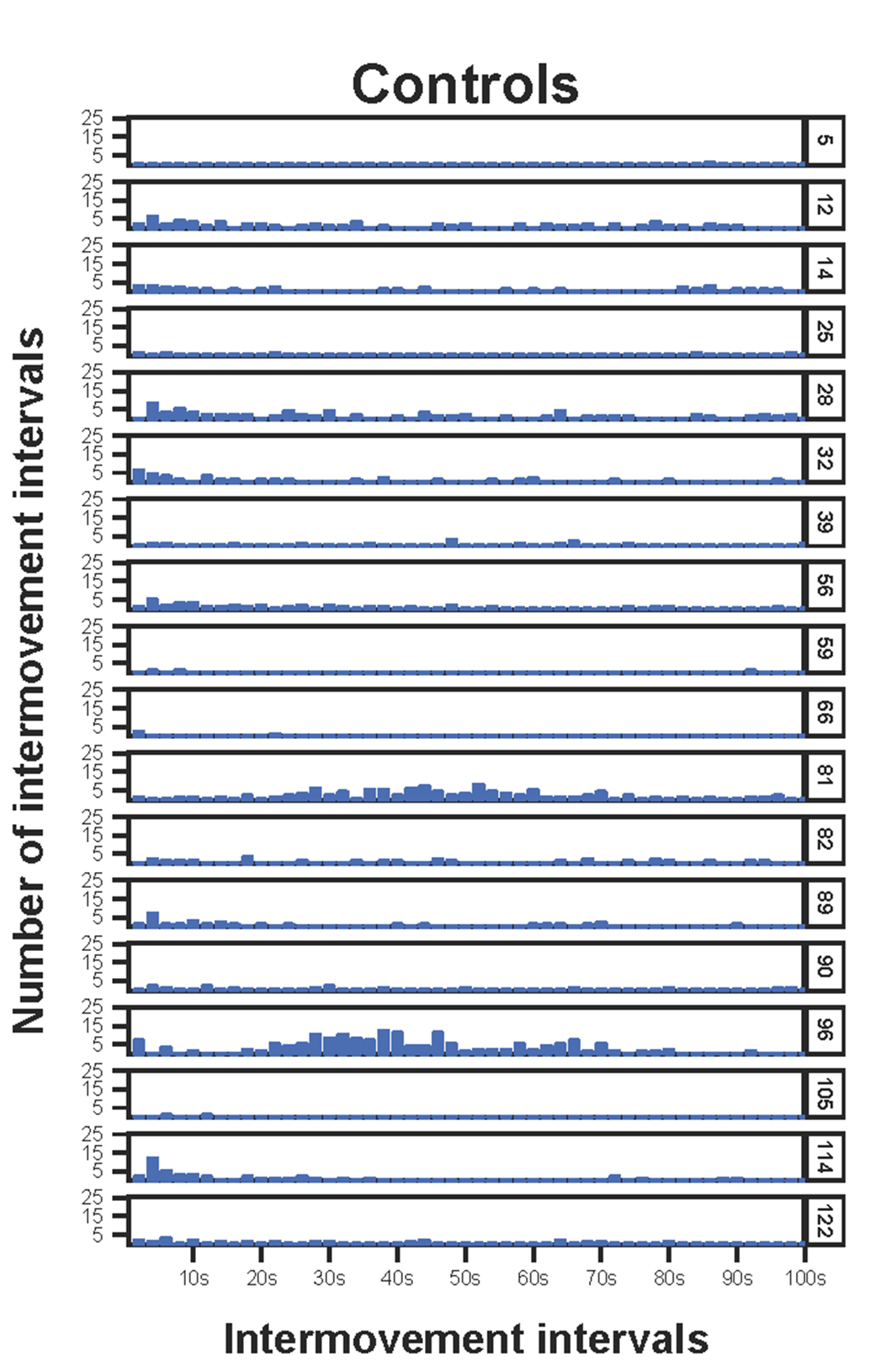

Supplement: Supplementary Image 3 — Distribution of intermovement intervals in control subjects. Healthy controls showed overall less prominent peaks in the left part of the graph, and only two subjects (KG081, KG096) showed any peak in the middle part of the graph, in particular in the 20–40 s range, which appeared less concentrated than in two ADHD subjects (see Supplementary Image 2). [file Image_3.tif]
